# Leukaemic pleural effusion as a manifestation of acute myeloid leukaemia: a case report and review of literature

**DOI:** 10.3332/ecancer.2014.397

**Published:** 2014-02-05

**Authors:** Amrita Duhan, Rajnish Kalra, Hemlata T Kamra, Anand Agarwal, Parveen Rana, Ruchi Agarwal, Sanjay Verma

**Affiliations:** BPS Government Medical College for Women, Khanpur, Sonepat, Haryana 131305, India

**Keywords:** acute myeloid leukaemia, pleural effusion, cytopathology

## Abstract

Haematologic malignancies such as acute and chronic leukaemias rarely present with or develop pleural effusion during their clinical course. We present a case of a young female who presented with unilateral pleural effusion and was diagnosed with haematologic malignancy on pleural fluid cytology. On further workup, a diagnosis of acute myeloid leukaemia was established. The patient was put on chemotherapy thereafter. This case clearly highlights the importance of cytopathology aids in making a diagnosis of rare and unusual presentation in haematologic malignancies.

## Introduction

Pleural effusion in patients with acute myeloid leukaemia (AML) is a rare occurrence and poorly understood. More rare is the detection of leukaemic cells in pleural effusion as the first morphologic manifestation of the disease [1]. Amongst the haematolymphoid malignancies, the most common disorders with pleural involvement are Hodgkin’s and non-Hodgkin’s lymphomas, with a frequency of 20–30%, especially if mediastinal involvement is present. Acute and chronic leukaemias are rarely accompanied by pleural effusions [2].

We report a case of a young female who was diagnosed with AML after a diagnostic thoracocentesis for pleural effusion.

## Case report

A 26-year-old female of low socio-economic status presented to the respiratory outpatient clinic with complaints of left-sided chest pain and dry cough for the preceding 20 days. She was afebrile. On examination, there was slight pallor but no lymphadenopathy, clubbing, jaundice, or pedal oedema. In the respiratory system examination, there was a dull note with decreased intensity of breath sounds in the left infrascapular region. There was mild hepatomegaly, but the spleen was not palpable. The clinical impression was anaemia with left-sided pleural effusion.

**Figure 1.**
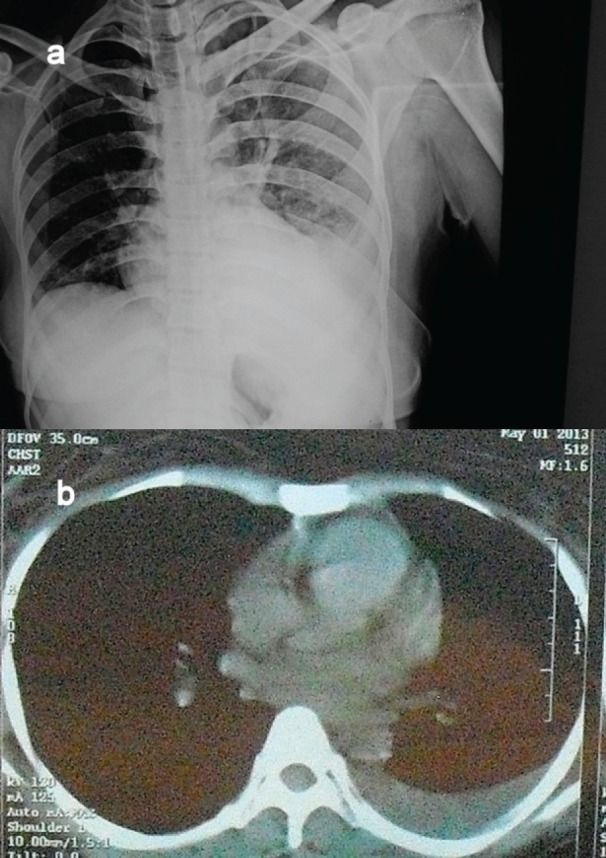
(a) and (b) Chest skiagram and CT scan of the patient, revealing left pleural effusion.

A chest X-ray and computed tomography (CT) scan of the patient showed moderate pleural effusion with basal collapse on the left side and no mediastinal lymphadenopathy (Figure 1).

**Figure 2.**
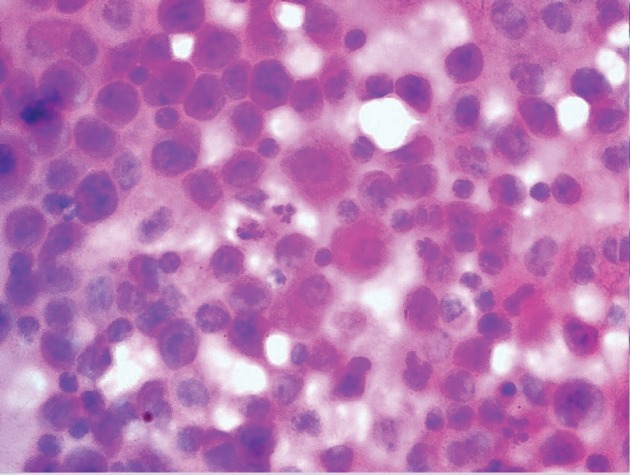
Cytospin-processed smear of pleural fluid revealing uniformly dispersed haematolymphoid blasts intermixed with mesothelial cells (H&E ×400).

**Figure 3.**
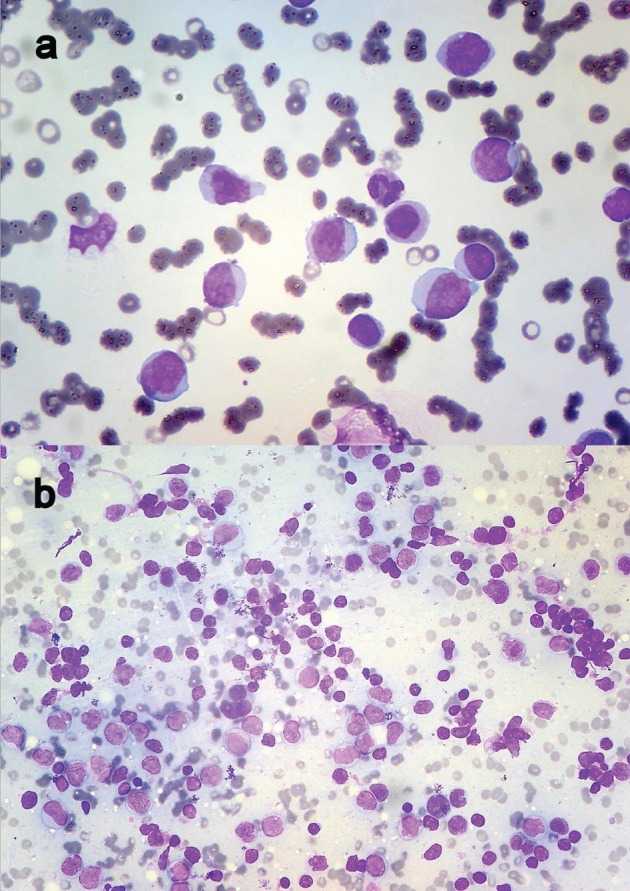
(a) Peripheral smear showing myeloblasts (Leishman ×400) and (b) FNAC of axillary lymph node revealing infiltration by myeloid blasts (Leishman ×200).

About 15 ml of blood-stained fluid was aspirated and sent for cytological analysis. The pleural fluid was exudate in nature, with protein 4.0 g/dl and glucose 42 mg/dl. The cytospin-processed smears revealed numerous uniformly dispersed and singly scattered haematolymphoid blasts against haemorrhagic background (Figure 2). Further workup of the patient was advised. A complete haemogram followed revealing haemoglobin—8.5 g/dl, total leucocyte count of 72,000/cumm with 82% blasts and decreased platelet count (Figure 3a). These blasts were Sudan positive. A diagnosis of AML-M4 was suggested, which was confirmed on bone marrow (BM) aspiration.

She was immediately put on induction chemotherapy (cytarabine, daunorubicin, and cladribine—three drug regimen) but showed minimal improvement. During the course, she developed axillary lymphadenopathy, aspiration of which revealed infiltration by myeloid blasts (Figure 3b). Her cytogenetic analysis could not be done because of financial constraints and the patient succumbed to the illness.

## Discussion

AML generally presents with signs and symptoms due to pancytopenia like fatigue, haemorrhage, and infections. Extramedullary involvement may accompany medullary involvement at the beginning or may occur as a complication. Hepatosplenomegaly, leukaemia cutis, lymphadenopathy, bone pain, granulocytic sarcomas, and gingival and central nervous involvement may occur. Both acute and chronic leukaemias rarely present with or are accompanied by pleural effusion [3]. Most cases reported in the literature concern acute lymhocytic leukaemia and very few cases of acute non-lymphocytic leukaemia [4]. After a thorough search of the literature through Internet using the keywords AML and pleural effusion via Google and PubMed search, only few cases/research articles have been found. The summary is discussed in the following paragraphs.

In the largest series of pleural procedures, Faiz *et al *observed 69 cases of pleural effusion associated with AML amongst 111 cases of acute leukaemia and MDS. The most frequent aetiology of effusion was infection (47%) followed by malignancy (36%). They concluded that survival in these patients is determined by the response to treatment of the haematologic malignancy [5]. Schmitt-Graff *et al *reported identification of focal leukaemic infiltrates as the initial manifestation of AML. Eight patients had myelodysplastic syndrome and over a twoyear period developed AML. Focal leukaemic infiltrates were localised in the skin, oral mucosa, lymph node, gastrointestinal tract, pleura, and retroperitoneum [6]. Boodosingh *et al *reported pleural effusion as the initial extramedullary manifestation of AML in a 66-year-old male who presented with worsening dyspnoea due to left-sided pleural effusion. On examination, submandibular, supraclavicular, axillary, and inguinal lymphadenopathy was present. Pleural fluid yielded exudative criteria with 62% leukaemic myeloblasts. Confirmation was done by BM biopsy [7]. Unilateral pleural effusion associated with AML has been reported by Fatih *et al *[3], Nieves-Nieves *et al * [8], and Ohe *et al *[9]. In the remote past, similar work has been published by Green *et al *[10], Gajwani [11], and Mital *et al *[12]. Although it is hard to evaluate the overall outcome of the patients with leukaemic effusions, a brief summary of the literature is given in Table 1.

Possible mechanisms of leukaemic pleural effusion in patients with AML include

(a) extramedullary proliferation of a quiescent leukaemic clone with subsequent metastasis to the BM;
(b) a subclinical marrow relapse, undetected by standard methods with consequent seeding to extramedullary sites. In our case, this might be the possible aetiology.

In leukaemic patients, other causes responsible for the presence of pleural effusion include bacterial or viral infections, other disseminated solid tumours, and complication of chemotherapy [5]. In most of the cases, the pleural fluid responds to the treatment of the primary disease, whereas the resistant or the relapsing cases may necessitate a pleurodesis [2].

The prognostic significance of the presence of pleural effusion at diagnosis in patients with acute leukaemia is not easy to determine. Some authors sustain that it does not affect the rate of remission and survival, while others report a worse prognosis [5]. The review of literature in the present study leads us to think that the presence of pleural effusion may indeed be a poor prognostic factor, as most cases sooner or later succumb to illness. The clinician should be alert for any signs of respiratory distress in AML patients at diagnosis, during chemotherapy and even on follow-up for an early therapeutic intervention. Cytogenetic abnormalities with poor prognosis include complex karyotype, -5, del(5q), -7, or abnormality of 3q [4]. The cytogenetic analysis of our patient was not done because of his poor socio-economic status; however, high suspicion of an abnormality with poor prognosis arises because of failure to respond to treatment.

In the treatment, pleural effusion usually disappears after induction chemotherapy, resulting in direct improvement of symptoms. If remission is not achieved, recurrence is almost inevitable, with many cases presenting with respiratory failure due to massive fluid accumulation. In such cases, intrapleural chemotherapy or chemical sclerosis is indicated [5].

## Conclusion

Most patients with a malignant effusion have a known history of primary, but sometimes a positive effusion may be the first sign of an unsuspected malignancy. Hence, cytological examination of a serous effusion may offer the possibility of an early and accurate diagnosis by using minimal intervention permitting appropriate therapy.

## Figures and Tables

**Table 1.** A brief summary of AML cases who presented as pleural effusion, from the published literature.

S. No.	Study	Age/sex	Type of AML	Appearance of effusion	PBF for myeloid cells	Course of disease
1	Fatih *et al* [3]	50/M	AML-M1	As initial and only manifestation	Negative	Patient succumbed to illness
2	Nieves-Nieves *et al* [8]	66/M	–	As initial presentation	Positive	Patient succumbed to illness
3	Ohe *et al* [9]	51/M	–	As initial and only manifestation	Negative	Complete remission in eight months follow-up
4	Agarwal *et al* [13]	45/M	AML-M2	As initial presentation	Positive	Patient succumbed to illness
5	Nayak *et al*[14]	68/M	AML-M4	As initial presentation	Positive	Patient succumbed to illness
6	Raina [2]	22/M	AML-M4	As initial presentation	Positive	Patient succumbed to illness
7	Chang *et al* [15] (three cases)	–	AML-M4	Two cases—as one of the complication of advanced AML One case—after diagnosis of AML	Positive Positive	Two cases—succumbed to illness One—survived
8	Park *et al* [16]	41/M	AML-M2	Recurrence after three years of bone marrow transplant for AML	Negative	Patient succumbed to illness
9	Present study	26/F	AML-M4	As initial presentation	Positive	Patient succumbed to illness
